# Supplementary figures and images for: Determining the effects of paternal obesity on sperm chromatin at histone H3 lysine 4 tri-methylation in relation to the placental transcriptome and cellular composition
Source: eLife. 2024 Nov 29;13:e83288. doi: 10.7554/eLife.83288 (PMC11717366; doi:10.7554/eLife.83288)

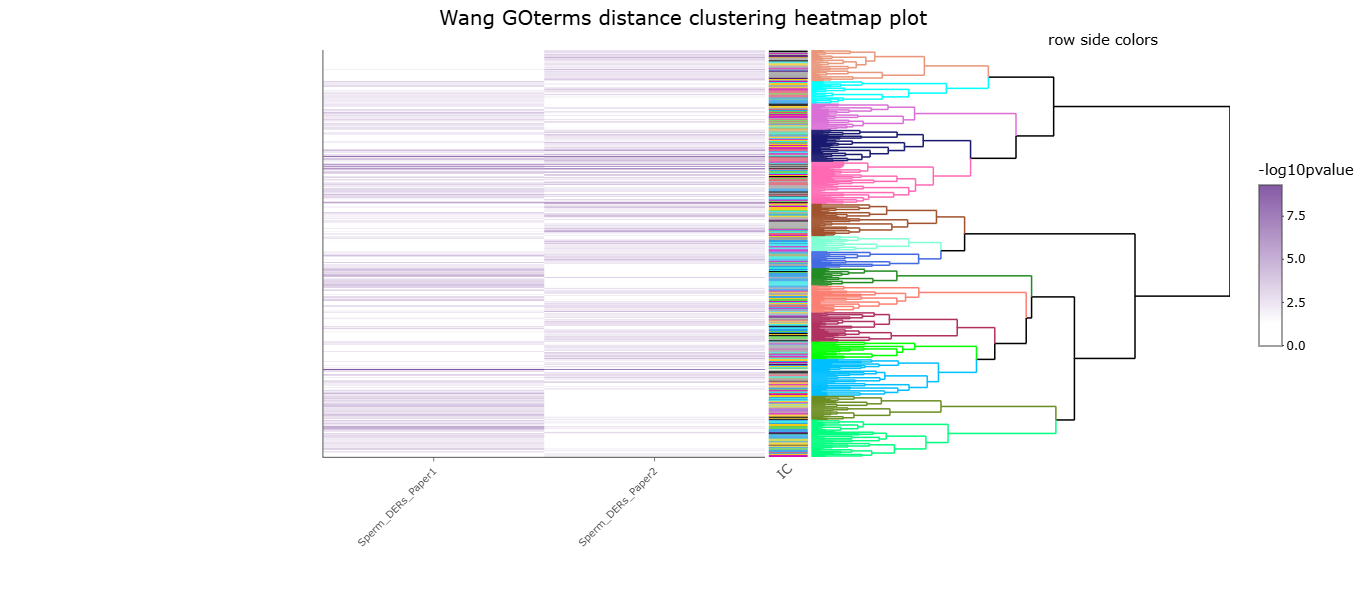

Supplement: Supplementary file 2. [file elife-83288-supp2.png]
